# Full-Genome Characterization of Simian T-Cell Leukemia Virus Type 1 Subtype b from a Wild-Born Captive *Gorilla gorilla gorilla* with T-Cell Lymphoma

**DOI:** 10.1128/genomeA.01117-17

**Published:** 2017-10-26

**Authors:** Ahidjo Ayouba, Annelies Michem, Martine Peeters, Francis Vercammen

**Affiliations:** a UMI233-TransVIHMI/INSERM U1175, Institut de Recherche Pour le Développement (IRD) and University of Montpellier, Montpellier, France; b Centre for Research and Conservation, Royal Zoological Society of Antwerp, Antwerp, Belgium

## Abstract

There are four lineages of primate T-cell lymphocytic viruses (human T-cell lymphocytic virus [HTLV]/simian T-cell lymphocytic virus [STLV]), which are further divided into subtypes. To date, there is only one full-length HTLV-1 subtype b genome available. Here, we report the genome of a new STLV-1 subtype b from a 43-year-old male gorilla with T-cell lymphoma.

## GENOME ANNOUNCEMENT

Human T-cell lymphotropic viruses (HTLVs) and simian T-cell lymphotropic viruses (STLVs) are collectively called primate T-cell lymphotropic viruses (PTLVs) (1). There are to date four known lineages of PTLVs, named PTLV-1 to PTLV-4, which infect a wide variety of humans and nonhuman primates (NHP) worldwide (2). PTLV lineages are divided into subtypes, and the four HTLV lineages have simian counterparts in monkeys and apes (3) corresponding to multiple cross-species transmissions from NHP to humans. The majority of human cases are due to the so-called cosmopolitan subtype of HTLV-1. Overall, HTLV-1 causes adult T-cell leukemia, a neurological disorder, or an inflammatory disease in less than 10% of infected patients (4). HTLV-2 is less pathogenic than HTLV-1 (5), and the clinical consequences of the few recorded HTLV-3 and HTLV-4 infections are currently unknown (6). In NHP, a few reported studies describe lymphomas/leukemias in captive gorillas (7) and macaques (8).

In Africa, infection with STLVs from the four lineages is endemic (9). Gorillas transmitted the so-called central African subtype of HTLV-1 (subtype b) and STLV-4 to humans (10). However, to date, only a single full genome of HTLV-1 subtype b has been characterized (11), and no full genomes of its primate ancestor have been characterized.

Here, we report the complete nucleotide sequence of an STLV-1 subtype b infecting a wild-born captive gorilla. Kumba (international studbook number 629) was a male western lowland gorilla (*Gorilla gorilla gorilla*) born wild in 1973 in southern Cameroon. At the age of 3 years, he was rescued after his mother was killed by hunters. He was transferred to a primate center and subsequently to Duisburg Zoo in Germany in 1976, then to the Amsterdam Zoo in 1999, and finally to the Antwerp Zoo in Belgium in 2002, where he died at the age of 43 years in 2016. Kumba did not suffer from any major disease between 2002 and November 2015. In December 2015, he became lethargic, lost his appetite, and was finally euthanized in the beginning of 2016. The necropsy showed a pancreas tumor, which was, according to the histopathology, a T-cell lymphoma. This observation prompted us to screen for STLV. By serology (INNO-LIA HTLV I/II score, Fujirebio), the sample was found positive for STLV-1, a result that was confirmed by a generic PTLV diagnostic PCR in the tax gene (12) performed on DNA extracted from a pancreas biopsy specimen by use of a Qiagen DNA tissue extraction kit. We determined the full genome of the novel STLV-1 sequence by PCR amplification and sequencing of subgenomic fragments of the genome. Generated sequences were assembled and corrected with SeqMan Pro 10.1.1 (DNAStar, Madison, WI, USA).

The full genome (STLV-1M10431) is 8,361 bp long and contains the pX gene in addition to the canonical *gag*, *pol*, and *env* genes common to all retroviruses. Interestingly, STLV-1M10431 encoded a functional HBZ protein in the reverse strand of the viral DNA, which was shown to play a role in the proliferation and maintenance of leukemic cells (13). Phylogenetic analyses showed that this isolate belongs to the PTLV-1 subtype b lineage throughout the whole genome. STLV-1M10431 is thus the first full-length genome of the subtype b STLV-1 sequence in NHP and only the second fully sequenced PTLV-1 subtype b.

### Accession number(s).

STLV-1M10431 was deposited in GenBank under the accession number MF622054.

## References

[1] Slattery JP, Franchini G, Gessain A 1999 Genomic evolution, patterns of global dissemination, and interspecies transmission of human and simian T-cell leukemia/lymphotropic viruses. Genome Res 9:525–540. 10400920

[2] Liégeois F, Lafay B, Switzer WM, Locatelli S, Mpoudi-Ngolé E, Loul S, Heneine W, Delaporte E, Peeters M 2008 Identification and molecular characterization of new STLV-1 and STLV-3 strains in wild-caught nonhuman primates in Cameroon. Virology 371:405–417. doi:10.1016/j.virol.2007.09.037. 17976676

[3] Ayouba A, Duval L, Liégeois F, Ngin S, Ahuka-Mundeke S, Switzer WM, Delaporte E, Ariey F, Peeters M, Nerrienet E 2013 Nonhuman primate retroviruses from Cambodia: high simian foamy virus prevalence, identification of divergent STLV-1 strains and no evidence of SIV infection. Infect Genet Evol 18:325–334. doi:10.1016/j.meegid.2013.04.015. 23612320

[4] Gessain A, Barin F, Vernant JC, Gout O, Maurs L, Calender A, de Thé G 1985 Antibodies to human T-lymphotropic virus type-I in patients with tropical spastic paraparesis. Lancet ii:407–410. doi:10.1016/S0140-6736(85)92734-5. 2863442

[5] Hirsch V, Allan J, Murthy K, Alford P, Slattery JP, O'Brien SJ, Franchini G, Koralnik IJ, Boeri E, Saxinger WC, Monico AL, Fullen J, Gessain A, Guo HG, Gallo RC, Markham P, Kalyanaraman V 1994 Phylogenetic associations of human and simian T-cell leukemia/lymphotropic virus type I strains: evidence for interspecies transmission. J Virol 68:2693–2707. 7908063 10.1128/jvi.68.4.2693-2707.1994 PMC236747

[6] Calattini S, Chevalier SA, Duprez R, Bassot S, Froment A, Mahieux R, Gessain A 2005 Discovery of a new human T-cell lymphotropic virus (HTLV-3) in central Africa. Retrovirology 2:30. doi:10.1186/1742-4690-2-30. 15882466 PMC1142341

[7] Blakeslee JR Jr., McClure HM, Anderson DC, Bauer RM, Huff LY, Olsen RG 1987 Chronic fatal disease in gorillas seropositive for simian T-lymphotropic virus I antibodies. Cancer Lett 37:1–6. doi:10.1016/0304-3835(87)90139-X. 2822228

[8] Homma T, Kanki PJ, King NW Jr., Hunt RD, O’Connell MJ, Letvin NL, Daniel MD, Desrosiers RC, Yang CS, Essex M 1984 Lymphoma in macaques: association with virus of human T lymphotrophic family. Science 225:716–718. doi:10.1126/science.6087453. 6087453

[9] Richard L, Mouinga-Ondémé A, Betsem E, Filippone C, Nerrienet E, Kazanji M, Gessain A 2016 Zoonotic transmission of two new strains of human T-lymphotropic virus type 4 in hunters bitten by a gorilla in central Africa. Clin Infect Dis 63:800–803. doi:10.1093/cid/ciw389. 27325689

[10] Gessain A, Mahieux R 2000 Epidemiology, origin and genetic diversity of HTLV-1 retrovirus and STLV-1 simian affiliated retrovirus. Bull Soc Pathol Exot 93:163–171. (In French.) 11030050

[11] Zanella L, Otsuki K, Marin MA, Bendet I, Vicente AC 2012 Complete genome sequence of central Africa human T-cell lymphotropic virus subtype 1b. J Virol 86:12451. doi:10.1128/JVI.02258-12. 23087114 PMC3486502

[12] Vandamme AM, Van Laethem K, Liu HF, Van Brussel M, Delaporte E, de Castro Costa CM, Fleischer C, Taylor G, Bertazzoni U, Desmyter J, Goubau P 1997 Use of a generic polymerase chain reaction assay detecting human T-lymphotropic virus (HTLV) types I, II and divergent simian strains in the evaluation of individuals with indeterminate HTLV serology. J Med Virol 52:1–7. 9131450

[13] Vernin C, Thenoz M, Pinatel C, Gessain A, Gout O, Delfau-Larue MH, Nazaret N, Legras-Lachuer C, Wattel E, Mortreux F 2014 HTLV-1 bZIP factor HBZ promotes cell proliferation and genetic instability by activating oncomirs. Cancer Res 74:6082–6093. doi:10.1158/0008-5472.CAN-13-3564. 25205102

